# Gradual deterioration of fatty liver disease to liver cancer *via* inhibition of AMPK signaling pathways involved in energy-dependent disorders, cellular aging, and chronic inflammation

**DOI:** 10.3389/fonc.2023.1099624

**Published:** 2023-03-02

**Authors:** Sha-Sha Meng, Hong-Wei Gu, Ting Zhang, Yu-Sang Li, He-Bin Tang

**Affiliations:** ^1^ Laboratory of Hepatopharmacology and Ethnopharmacology, School of Pharmaceutical Sciences, South-Central Minzu University, Wuhan, China; ^2^ Department of Pharmacy, Wuhan Mental Health Center, Wuhan, China

**Keywords:** fatty Liver, AMPK, inflammation, hypoxia, aging, tumor microenvironment, liver cancer

## Abstract

**Introduction:**

Hepatocellular carcinoma (HCC) is the most prevalent primary liver cancer kind. According to recent research, a fatty liver increases the risk of hepatocellular cancer. Nevertheless, the AMPK signaling pathway is crucial. In addition, 5’-AMP-activated protein kinase (AMPK) is strongly linked to alterations in the tumor microenvironment, such as inflammation, hypoxia, and aging. The objective of this study is to evaluate the impact of the AMPK signaling pathway on the progression of fatty liver to HCC.

**Methods:**

In this study, we established a mouse liver cancer model using high-fat diets and nano-nitrosamines (nano-DEN). In addition, we employed a transcriptomic technique to identify all mRNAs detected in liver samples at the 25th weekexpression of proteins linked with the LKB1-AMPK-mTOR signaling pathway, inflammation, aging, and hypoxia was studied in microarrays of liver cancer tissues from mice and humans. These proteins included p-AMPK, LKB1, mTOR, COX-2, β-catenin, HMGB1, p16, and HIF-1α.

**Results:**

Data were collected at different times in the liver as well as in cancerous and paracancerous regions and analyzed by a multispectral imaging system. The results showed that most of the genes in the AMPK signaling pathway were downregulated. *Prakk1* expression was upregulated compared to control group but downregulated in the cancerous regions compared to the paracancerous regions. *Stk11* expression was downregulated in the cancerous regions. *Mtor* expression was upregulated in the cancerous regions. During liver cancer formation, deletion of LKB1 in the LKB1-AMPK-mTOR signaling pathway reduces phosphorylation of AMPK. It contributed to the upregulation of mTOR, which further led to the upregulation of HIF1α. In addition, the expression of β-catenin, COX-2, and HMGB1 were upregulated, as well as the expression of p16 was downregulated.

**Discussion:**

These findings suggest that changes in the AMPK signaling pathway exacerbate the deterioration of disrupted energy metabolism, chronic inflammation, hypoxia, and cellular aging in the tumor microenvironment, promoting the development of fatty liver into liver cancer.

## Introduction

Primary liver cancer will be the third biggest cause of cancer deaths worldwide in 2020. There will be approximately 906,000 new cases of liver cancer and 830,000 deaths worldwide in 2020, with the number of deaths close to the number of new cases (1). Nonalcoholic fatty liver disease (NAFLD) has a global prevalence of 25%, is one of the primary causes of cirrhosis and HCC, and is a very common chronic disease believed to be linked to metabolic dysfunction (2). NAFLD is an increasingly significant risk factor for liver cancer development (3). It is estimated that about 1/4 of the world’s population has NAFLD, and the incidence of NAFLD-related HCC is increasing at an annual rate of 9% (4).

NAFLD is a prevalent cause of cryptogenic cirrhosis and HCC of unknown origin, and the development of NAFLD-HCC is linked to alterations in inflammatory and tumor-related signaling pathways (5). However, the specific mechanism by which NAFLD develops into HCC remains unclear. Therefore, it is vital to identify new treatment targets to monitor the evolution of liver cancer that originates from a fatty liver. It has been shown that p-AMPK inhibits NAFLD and disrupts fat formation in HCC (6, 7). AMPK is an energy receptor that senses glucose availability independently of changes in adenine nucleotides (8). Activation of the AMPK signaling pathway inhibits the glycolysis pathway, which in turn inhibits the progression of liver cancer (9). AMPK is a key molecule in the regulation of biological energy metabolism and plays an important role in this process.

Chronic liver inflammation is associated with tumorigenesis (10). In most cases, liver cancer occurs in the context of chronic liver inflammation caused by viral hepatitis and alcoholic or nonalcoholic steatohepatitis (11). Deregulated Wnt/β-catenin signaling is one of the main genetic alterations in human liver cancer (12). In addition, there is growing evidence that abnormalities in Wnt/β-catenin signaling promote the development and progression of liver cancer (13).

In the majority of instances, liver cancer develops in the context of chronic liver inflammation resulting from viral hepatitis and alcoholic or nonalcoholic steatohepatitis. HCC exists in an immunosuppressed milieu that favors tumor evasion, and hypoxia can influence intercellular communication in the microenvironment of the tumor (14, 15). Hypoxia is a key factor in the induction of transcription of *HIF1A*, HIF-1α protein accumulates to promote angiogenesis (16). It is currently believed that HIF-1 and AMPK are important regulators of the switch between reprogramming and oxidative metabolism (17).

Cellular senescence is a program that prevents the malignant transformation of senescent cells following oncogenic pathway activation and DNA damage. Senescent cells are metabolically active and secrete cytokines and chemokines that shape the function and composition of their microenvironment (18). Increased expression of the p16 oncogene associated with cellular senescence leads to senescence. In contrast, cancer cell overcome this effect by inactivating the gene through homologous deletion or hypermethylation (19).

The specific mechanism by which nonalcoholic fatty liver develops into HCC remains unclear. AMPK, inflammation-related β-catenin, COX-2, HMGB1, hypoxia-related HIF-1α, and cellular senescence-related p16 are all important in the development of liver cancer. Therefore, this paper explores the influence and mechanism of the AMPK signaling pathway on the tumor microenvironment during the progression from fatty liver to liver cancer. It can provide a reference for the treatment of liver cancer that develops from fatty liver.

## Materials and methods

### Reagents

The following drug was used in this experiment: diethylnitrosamine (DEN; Tokyo Chemical Industry Co., Ltd., Tokyo, Japan). The antibodies used in this study included antibodies against β-catenin, COX-2, HMGB1, p16, HIF-1α (Abcam Inc., Cambridge, MA, UK), anti-phospho-AMPKα (pThr172; Sigma Chemical Co., St. Louis, MO, USA), LKB1 and mTOR (ABclonal Technology, Wuhan, China). The instruments used in the experiment were as follows: a multispectral quantitative instrument (Cambridge Research & Instrumentation, Inc., Woburn, MA, USA, model Nuance), a fusion instrument (Servicebio, Wuhan, China, Model F-16), and a sampling handle (Servicebio, Wuhan, China, model SH-20).

### Animal care

Before the initiation of experiments, 25 male Kunming mice (18-22 g) from the Hubei Experimental Animal Center were acclimatized for 7 days under SPF conditions. The animals were reared in a temperature-controlled animal feeding center (20-25°C) with a 12-hour light-dark cycle. Kunming mice were randomly divided into two groups: control (n = 5), model (n = 20). The control group was fed a normal diet, while the model group was fed a high-fat diet. During the experiment, the animals in the model group were injected intraperitoneally with the chemical poison nano-DEN (16.5 mg/kg) weekly (20).

The control group did not have any treatment, and the mice were sacrificed on the first day of the experiment. When the mice were sacrificed, the livers were collected immediately and collected for histological and protein analysis. Twenty mice from the model group were sacrificed, and liver tissues were collected from mice at the 10^th^, 15^th^, 20^th^, and 30^th^ week, with five mice sacrificed each week. Then, protein levels were assessed by immunohistochemical staining. The livers of the control, HF (high-fat), ND (nano-DEN), and HFND (co-exposure of high-fat diet and nano-DEN) group mice were transcriptomic analyzed at the 25^th^ week. The livers of mice in the HFND group were divided into HFNDC (high fat-diet and nano-DEN’s co-exposure to cancerous regions) and HFNDP (high fat-diet and nano-DEN’s co-exposure to paracancerous regions). The data that support the findings of this study have been deposited into CNGB Sequence Archive (21) of China National GeneBank DataBase (22) with accession number CNP0003645. For hepatocellular steatosis, specimens were scored 0 – 3 points. 0 points: no fat; 1 point: steatosis occupying < 33% of the hepatic parenchyma; 2 points: 34 – 66% of the hepatic parenchyma; and 3 points: more than 66% of the hepatic parenchyma (23).

The protocols for the management and usage of animals and experiments for this study were performed by the Laboratory Animal Society of the Research Facilities Committee. All research on mice was approved by the Animal Experiment Ethics Committee of South-Central Minzu University, Wuhan, China (permit number: 2018-SCUEC-AEC-010).

### Human samples and their tissue micro-array (TMA) analysis

Liver tissue samples from 30 liver cancer patients were obtained from the Key Laboratory of Chinese Internal Medicine of MOE of the Beijing Dongzhimen Hospital of Beijing University of Chinese Medicine. The cancerous regions were obtained from carcinoma tissues and reviewed by professional pathologists. The paracancerous regions were obtained from normal tissues over 3 cm from the borderline of the cancerous regions in the same fixed slice. The collection and follow-up manipulation of all pathological tissue samples were approved by the Committee on the Ethics of Experiments of South-Central Minzu University in China (Permit Number: 2017-SCUEC-MEC-007).

The tissues were extracted using a tissue chip handle (2 mm) and then put into a 96-well paraffin mold (2 mm). After that, the mold was heated to keep the tissue flat. The remaining paraffin was added to the mold to fill the gaps between the holes. The TMA was made after the tissue had cooled (24).

### Immunohistochemical analysis

The sliced sections were immersed in xylene followed by absolute alcohol for dewaxing and dehydration. After incubation in 3% H_2_O_2_ hydrogen peroxide, for antigen retrieval, sections were heated in a microwave oven for 10 min in 0.01 M citric saline (pH = 6.0). After blocking in an oven at 37°C for 1 hour with the appropriate serum albumin, the sections were incubated with anti-phospho-AMPKα (pThr172; 1:100 dilution), LKB1 (1:100 dilution), mTOR (1:100 dilution), β-catenin (1:200 dilution), COX-2 (1:200 dilution), HMGB1 (1:200 dilution), p16 (1:200 dilution) and HIF-1α (1:100 dilution) antibodies overnight. After the corresponding secondary antibodies were directly applied to the tissue sections and incubated at 37°C for 1 hour, they were confirmed to be microscopic positive by staining with diaminobenzidine (DAB) chromophore followed by counterstaining with hematoxylin. Finally, the slices were sequentially dehydrated and sealed by gradient alcohol and xylene. As a negative control, 1% bovine serum albumin (BSA) was used to replace the primary antibody on sections that were proven to be positive for p-AMPKα (pThr172), LKB1, mTOR, β-catenin, COX-2, HMGB1, p16 and HIF-1α in the present experiments. Then, imaging analysis of sections was performed by using a Nikon 50i light microscope imaging system (Nikon).

### Statistical analysis

Prism 9 (GraphPad Software) was used to process the data. Bar charts were constructed, and statistical analyses were performed. The quantitative immunohistochemical staining results showed positive expression intensity. All data are presented as the mean ± SEM. A paired *t*-test was used to compare the differences between the two groups in the cancerous and paracancerous regions, and an unpaired *t*-test was used to compare the differences between the groups at different times and the control group. *, *p* < 0.05, **, *p* < 0.01, and ***, *p* < 0.001 were considered statistically significant, very significant, and extremely significant, respectively, and *p* > 0.05 was considered not significant.

## Results

### Analysis of differential gene expression of the AMPK signaling pathway in the livers of mice with liver cancer

**Figure 1:**
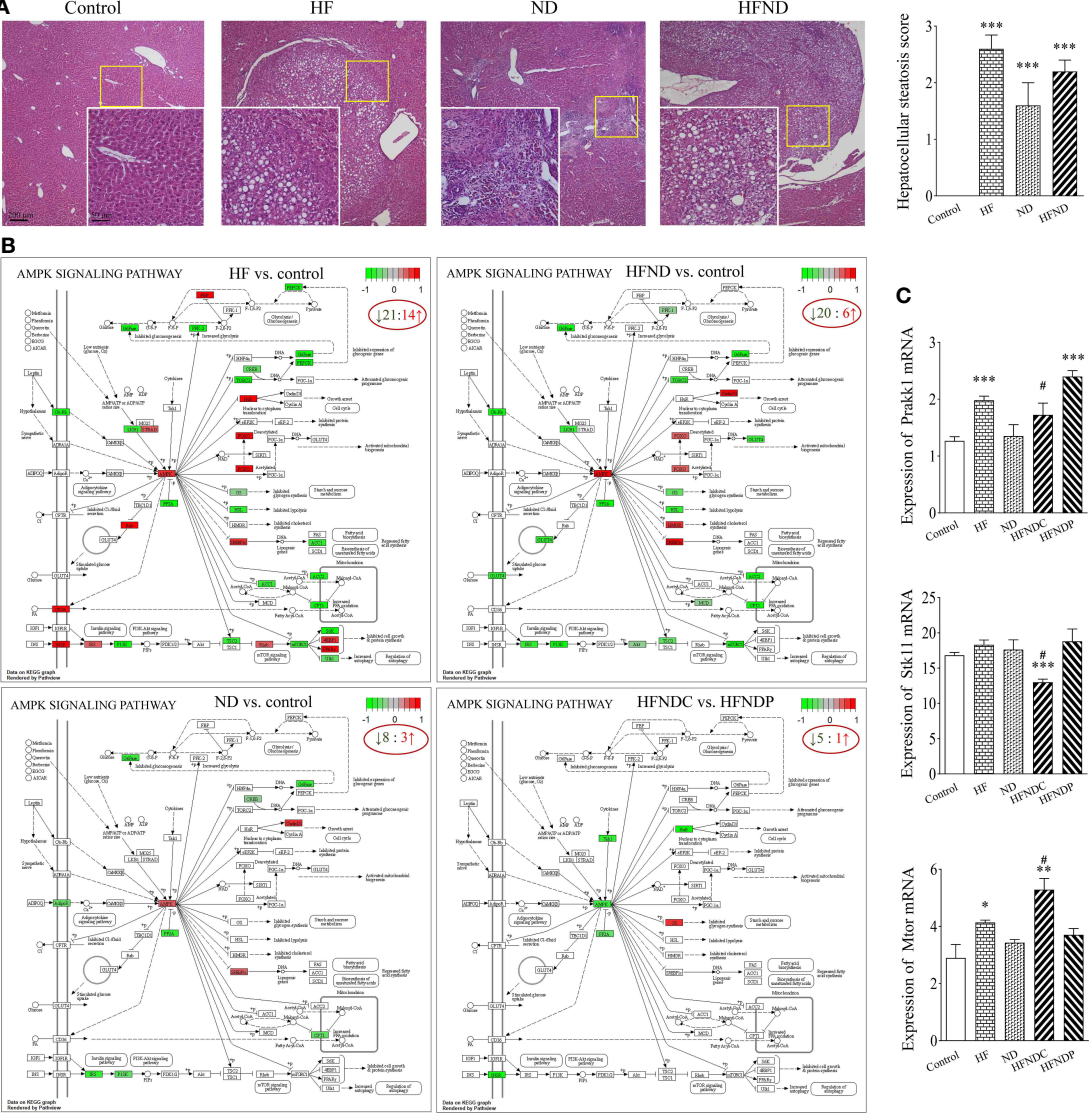
General view of mouse livers and KEGG pathways: AMPK signaling pathway and mRNA expression of *Prakk1*, *Stk11*, and *Mtor*. **(A)** General view of the liver samples and Hepatocellular steatosis score. **(B)** KEGG pathways: Changes in AMPK signaling pathway genes in different groups. **(C)** Statistical quantification of the mRNA levels of *Prakk1*, *Stk11*, and *Mtor* in the livers of mice. Upregulated genes are marked in red, and downregulated genes are marked in green. The experimental data are expressed as the mean ± SEM. Compared to the control, *p < 0.05; **p < 0.01; ***p < 0.001. Compared to HFNDP, ^#^ *p* < 0.05. Scale bar, 50 µm and 200 µm.

In **Figure 1A**, we were able to observe the pathological structural changes in the livers of different groups of mice from several representative images. In the control group, the liver structure was intact and clear, while the HF group showed a large number of fat vacuoles (2.60 ± 0.24, *p* < 0.001). Multiple inflammatory sites and abnormal aggregation of cells with partial fatty degeneration (1.60 ± 0.40, *p* < 0.001) could be seen in the ND group. In the HFND group, there was an accumulation of abnormal cells with enlarged nuclei and multiple points of inflammation in the cancerous regions, which were distinctly different from normal liver tissue and showed more steatosis (2.20 ± 0.20, *p* < 0.001). In our earlier research, it was found that only mice treated with nano-DEN showed significant tumor nodules at the 25^th^ to 35^th^ week (25). However, very obvious tumor nodules already appeared at the 25^th^ week after co-exposure of high-fat diet and nano-DEN, which significantly accelerated the carcinogenesis process.

We also analyzed genes with differential expression in the AMPK signaling pathway in the livers of mice with liver cancer. We found that 60.0% (HF vs. control; 21/35), 72.7% (ND vs. control; 8/11), 76.9% (HFND vs. control; 20/26), and 83.3% (HFNDC vs. HFNDP; 5/6) of the genes in the AMPK signaling pathway were downregulated (**Figure 1B**).

According to **Figure 1C**, the expression of *Prakk1* was upregulated in the HF group (1.98 ± 0.07, *p* < 0.001), ND group (1.35 ± 0.20, *p* > 0.05), and the HFNDC group (1.73 ± 0.18, *p* > 0.05) compared to the control group (1.27 ± 0.07), while the expression was downregulated in the HFNDC compared to the HFNDP (2.40 ± 0.09, *p* < 0.05). *Stk11* expression was upregulated in the HF group (18.32 ± 0.66, *p* > 0.05) and ND group (17.63 ± 1.38, *p* > 0.05) compared to the control group (16.81 ± 0.41). Conversely, *Stk11* expression was downregulated in the HFNDC (13.01 ± 0.40, *p* < 0.001), while cancerous regions was downregulated compared to the HFNDP (18.81 ± 1.56, *p* < 0.05). The expression of *Mtor* was upregulated in the HF group (4.14 ± 0.08, *p* < 0.05), ND group (3.43 ± 0.11, *p* > 0.05), and the HFNDC (5.29 ± 0.35, *p* < 0.01) compared to the control group (2.90 ± 0.47), while HFNDC was also significantly upregulated compared to the HFNDP (3.71 ± 0.20, *p* < 0.05). So we can see that the AMPK signaling pathway and related genes show more severe changes in the HFND group than in the ND group during the same period.

#### Downregulation of LKB1 and p-AMPK expression and upregulation of mTOR expression in liver tissues of mice with liver cancer

**Figure 2:**
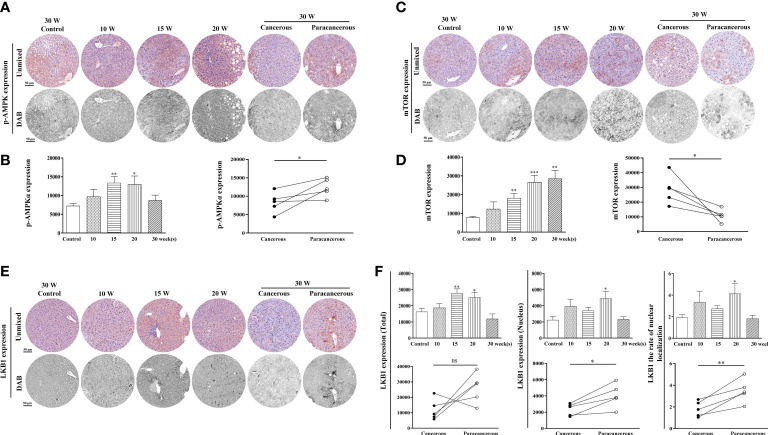
Immunohistochemical staining of p-AMPK, mTOR, and LKB1 proteins in mouse livers at different time points and their quantitative representative images. **(A)** Representative images of immunohistochemical staining of p-AMPK in mouse livers during liver carcinogenesis. **(B)** Quantitative multispectral image of the p-AMPK protein. **(C)** Representative images of immunohistochemical staining of mTOR in mouse livers during liver carcinogenesis. **(D)** Quantitative multispectral image of the mTOR protein. **(E)** Representative images of immunohistochemical staining of LKB1 in mouse livers during liver carcinogenesis. **(F)** Quantitative multispectral image of the LKB1 protein. The experimental data are expressed as the mean ± SEM. Compared to the control, *p < 0.05; **p < 0.01; ***p < 0.001. Compared to paracancerous regions, *p < 0.05. Scale bar, 50 µm. ns, not statistically.

The expression levels of LKB1, p-AMPK, and mTOR proteins in the liver tissues are summarized in **Figure 2**. The experimental results were as follows: in **Figures 2A, B**, compared with the control group, p-AMPK expression was continuously increased from the 10^th^ to the 20^th^ week, and the expression in the cancerous regions was decreased at the 30^th^ week. Compared with the paracancerous regions (12316 ± 1115), the expression in the cancerous regions (8715 ± 1395) was downregulated (downregulated to 71% of the paracancerous regions, *p* < 0.05). At the 20^th^ week, a large number of fat vacuoles appeared in the liver tissues of mice, the expression of p-AMPKα was abnormal with time, and liver metabolism was disturbed (**Figure 2A**).

As shown in **Figures 2C, D**, compared with the control group, the expression levels of mTOR continuously increased from the 10^th^ to the 30^th^ week. Compared with the paracancerous regions (10868 ± 1867), the expression in the cancerous regions (28626 ± 4396) was upregulated (upregulated to 263% of that in the paracancerous regions, *p* < 0.05).

As shown in **Figures 2E, F**, compared with the control group, the total expression of LKB1 was continuously increased from the 10^th^ to the 15^th^ week, and decreased at the 20^th^ week. The expression in the cancerous regions was significantly decreased at the 30^th^ week. Compared with the paracancerous regions (25991 ± 4322), the expression in the cancerous regions (11925 ± 3046) was downregulated (downregulated to 46% of that in the paracancerous regions, *p* > 0.05). Compared with the nuclear expression of LKB1 in the paracancerous regions (4048 ± 649), cancerous regions (2331 ± 331) was downregulated (downregulated to 58% of that in the paracancerous regions, *p* < 0.05). Compared with the paracancerous regions (3.50 ± 0.48), the nucleation rate in the cancerous regions (1.82 ± 0.32) was downregulated (downregulated to 52% of that in the paracancerous regions, *p* < 0.01). LKB1 may be decompensated and increased in the process of liver cancer, and its expression in cancer tissues is absent after carcinogenesis. LKB1 is an upstream kinase of AMPK and regulates AMPK phosphorylation. Therefore, p-AMPK increases first and decreases after carcinogenesis, leading to increased expression of mTOR.

#### Expression of the inflammation-related proteins β-catenin, COX-2, and HMGB1 was upregulated in liver tissues of mice with liver cancer

**Figure 3:**
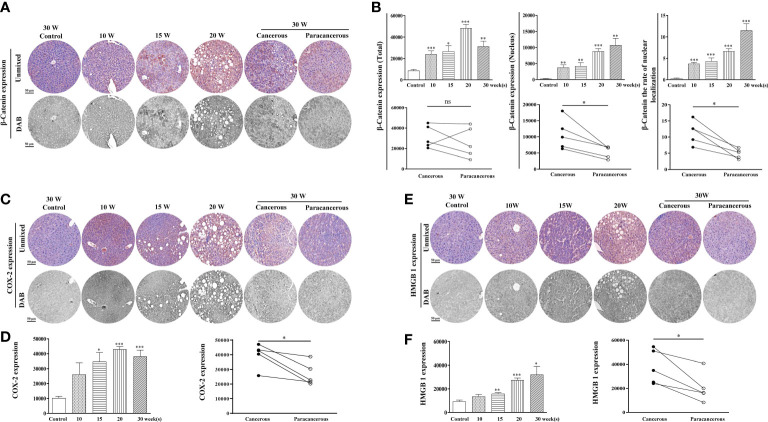
Immunohistochemical staining of β-catenin, COX-2, and HMGB1 proteins in mouse livers at different time points and their quantitative representative images. **(A)** Representative images of immunohistochemical staining of β-catenin in mouse livers during liver carcinogenesis. **(B)** Quantitative multispectral image of the β-catenin protein. **(C)** Representative images of immunohistochemical staining of COX-2 in mouse livers during liver carcinogenesis. **(D)** Quantitative multispectral image of the COX-2 protein. **(E)** Representative images of immunohistochemical staining of HMGB1 in mouse livers during liver carcinogenesis. **(F)** Quantitative multispectral image of the HMGB1 protein. The experimental data are expressed as the mean ± SEM. Compared to the control, *p < 0.05; **p < 0.01; ***p < 0.001. Compared to paracancerous regions, *p < 0.05; **p < 0.01. Scale bar, 50 µm. ns, not statistically.

The expression levels of β-catenin, COX-2, and HMGB1 in the liver tissues are illustrated in **Figure 3**. We found that compared with the control group, the expression of β-catenin increased from the 10^th^ to the 20^th^ week, and decreased at the 30^th^ week in the cancerous regions (**Figures 3A, B**). In addition, compared with the paracancerous regions (25852 ± 6759), the expression of the cancerous regions (31316 ± 4932) was upregulated (upregulated to 121% of that in the paracancerous regions, *p* > 0.05). Compared with the nuclear expression of β-catenin in the paracancerous regions (5343 ± 831), cancerous regions (10748 ± 2127) was upregulated (upregulated to 201% of that in the paracancerous regions, *p* < 0.05). Compared with the paracancerous regions (4.92 ± 0.67), the nucleation rate of the cancerous regions (11.48 ± 1.60) was upregulated (upregulated to 233% of that in the paracancerous regions, *p* < 0.05).

As shown in **Figures 3C, D**, compared with the control group, COX-2 expression continuously increased from the 10^th^ to the 30^th^ week, and the highest expression was observed at the 20^th^ week. Compared with the paracancerous regions (26686 ± 3473), the expression in the cancerous regions (39816 ± 3673) was upregulated (upregulated to 149% of that in the paracancerous regions, *p* < 0.05). As shown in **Figures 3E, F**, compared with the control group, HMGB1 expression continuously increased from the 10^th^ week to the 30^th^ week. Compared with the paracancerous regions (20295 ± 5462), the expression in the cancerous regions (37954 ± 6362) was upregulated (upregulated to 187% of that in the paracancerous regions, *p* < 0.05).

#### Downregulation of aging factor p16 expression and upregulation of hypoxic factor HIF-1α expression in liver tissues of liver cancer mice

**Figure 4:**
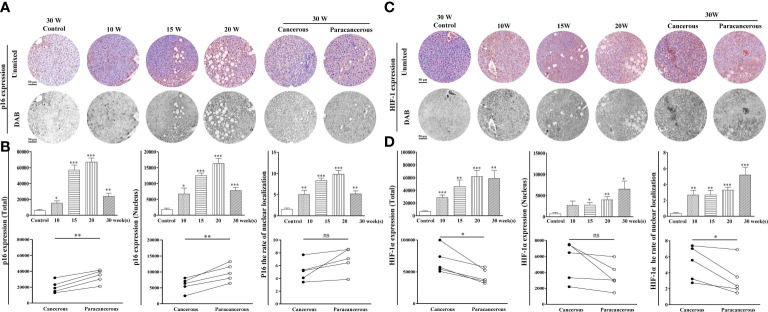
Immunohistochemical staining of p16 and HIF-1α proteins in mouse livers at different time points and their quantitative representative images. **(A)** Representative images of immunohistochemical staining of p16 in mouse livers during liver carcinogenesis. **(B)** Quantitative multispectral image of the p16 protein. **(C)** Representative images of immunohistochemical staining of HIF-1α in mouse livers during liver carcinogenesis. **(D)** Quantitative multispectral image of the HIF-1α protein. The experimental data are expressed as the mean ± SEM. Compared to the control, *p < 0.05; **p < 0.01; ***p < 0.001. Compared to paracancerous regions, *p < 0.05. Scale bar, 50 µm. ns, not statistically.

**Figure 4** shows the expression levels of p16 and HIF-1α in the liver tissues. As shown in **Figures 4A, B**, compared with the control group, the total expression of p16 continuously increased from the 10^th^ to the 20^th^ week, and the expression in the cancerous regions decreased at the 30^th^ week. Compared with the paracancerous regions (33703 ± 3711), the expression in the cancerous regions (20421 ± 3377) was downregulated (downregulated to 61% of that in the paracancerous regions, *p* < 0.01). Compared with the nuclear expression of p16 in the paracancerous regions (9668 ± 1272), cancerous regions (5828 ± 971) was downregulated (downregulated to 60% of that in the paracancerous regions, *p* < 0.01). Compared with the paracancerous regions (6.90 ± 0.86), the nucleation rate of the cancerous regions (5.14 ± 0.73) was downregulated (downregulated to 75% of that in the paracancerous regions, *p* > 0.05).

According to **Figures 4C, D**, compared with the control group, the total expression of HIF-1α was continuously increased from the 10^th^ to the 30^th^ week, and compared with the expression in the paracancerous regions (43064 ± 5037), cancerous regions (67346 ± 9082) was upregulated (upregulated to 156% of that in the paracancerous regions, *p* < 0.05). Compared with the nuclear expression of HIF-1α in the paracancerous regions (3551 ± 763), cancerous regions (5400 ± 1104) was upregulated (upregulated to 152% of that in the paracancerous regions, *p* > 0.05). Compared with the paracancerous regions (3.24 ± 0.98), the nucleation rate of the cancerous regions (5.20 ± 0.95) was upregulated (upregulated to 161% of that in the paracancerous regions, *p* < 0.05).

### Downregulation of LKB1 and p-AMPK expression and upregulation of mTOR expression in cancerous regions of liver cancer patientsns

**Figure 5:**
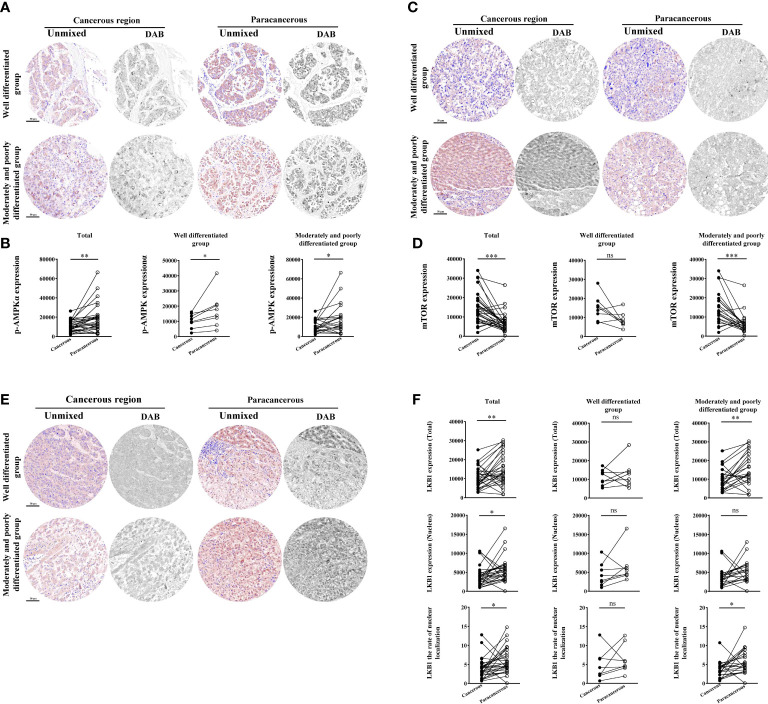
Immunohistochemical staining of p-AMPK, mTOR, and LKB1 proteins in liver cancer tissues and their quantitative images. **(A)** Representative image of immunohistochemical staining of the p-AMPK protein in liver tissues. **(B)** Quantitative multispectral image of the p-AMPK protein. **(C)** Representative image of immunohistochemical staining of the mTOR protein in liver tissues. **(D)** Quantitative multispectral image of the mTOR protein. **(E)** Representative image of immunohistochemical staining of the LKB1 protein in liver tissues. **(F)** Quantitative multispectral image of the LKB1 protein. The experimental data are expressed as the mean ± SEM. Compared to paracancerous regions, *p < 0.05; **p < 0.01; ***p < 0.001. Scale bar, 50 µm. ns, not statistically.

As shown in **Figures 5A, B**, compared with the paracancerous regions (18103 ± 2668), the expression of p-AMPK in the cancerous regions (10687 ± 1062) was downregulated (downregulated to 59% of that in the paracancerous regions, *p* < 0.01). Moreover, compared with the well-differentiated group, the expression of p-AMPK in the cancerous regions of the moderately and poorly differentiated group was downregulated more significantly (*p* < 0.05).

According to **Figures 5C, D**, the expression of mTOR was significantly upregulated in the cancerous regions (14624 ± 1461) compared with the paracancerous regions (7194 ± 920; upregulated to 203% of that in the paracancerous regions, *p* < 0.001). However, there was no significant difference in tissues expression between the cancerous and paracancerous regions in the well-differentiated group. However, the expression of mTOR was more significantly upregulated in the cancerous regions of the intermediate and moderately and poorly differentiated group (*p* < 0.001) compared to the well-differentiated group.

However, compared with the paracancerous regions (14802 ± 1524), the total expression of LKB1 in the cancerous regions (10348 ± 1007) was downregulated (downregulated to 70% of that in the paracancerous regions, *p* < 0.01). Compared with the well-differentiated group, the total expression in the moderately and poorly differentiated group was downregulated more significantly (*p* < 0.01). Compared with the paracancerous regions (5595 ± 594), the nuclear expression of LKB1 in the cancerous regions (3989 ± 474) was downregulated (downregulated to 71% of that in the paracancerous regions, *p* < 0.05). Compared with the paracancerous regions (5.96 ± 0.60), the nucleation rate in the cancerous regions (3.91 ± 0.49) was downregulated (downregulated to 66% of that in the paracancerous regions, *p* < 0.05; **Figures 5E, F**).

### Expression of the inflammation-related proteins β-catenin, HMGB1, and COX-2 was upregulated in the cancerous regions of liver cancer patients

**Figure 6:**
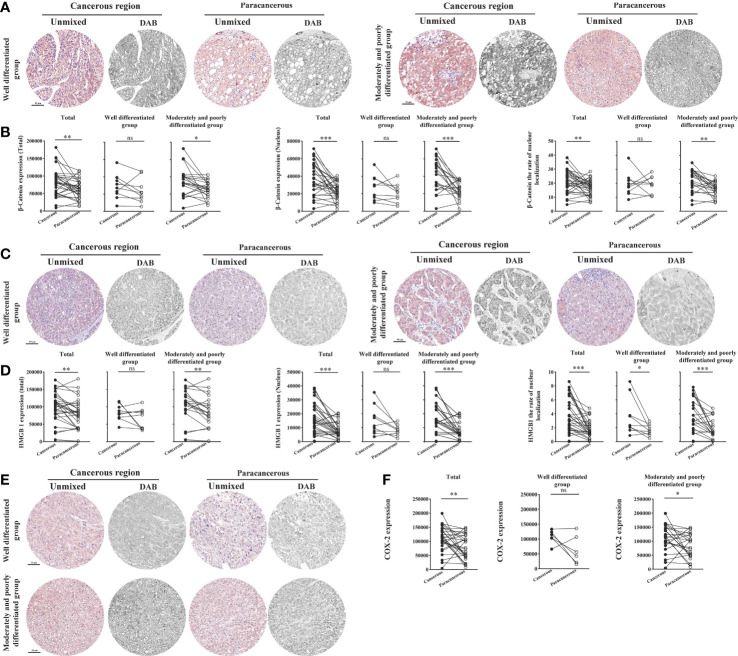
Immunohistochemical staining of β-catenin, HMGB1, and COX-2 proteins in liver cancer tissues and their quantitative images. **(A)** Representative image of immunohistochemical staining of the β-catenin protein in liver tissues. **(B)** Quantitative multispectral image of the β-catenin protein. **(C)** Representative image of immunohistochemical staining of the HMGB1 protein in liver tissues. **(D)** Quantitative multispectral image of the HMGB1 protein. **(E)** Representative image of immunohistochemical staining of the COX-2 protein in liver tissues. **(F)** Quantitative multispectral image of the COX-2 protein. The experimental data are expressed as the mean ± SEM. Compared to paracancerous regions, *p < 0.05; **p < 0.01; ***p < 0.001. Scale bar, 50 µm. ns, not statistically.

As illustrated in **Figures 6A, B**, we found that the total expression of β-catenin in the cancerous regions, the nuclear expression, and the nucleation rate were significantly upregulated compared with those in the paracancerous regions. Compared with the paracancerous regions (61827 ± 4985), the total expression of β-catenin in the cancerous regions (82403 ± 7283) was upregulated (upregulated to 133% of that in the paracancerous regions, *p* < 0.01). Compared with the well-differentiated group, the total expression in the moderately and poorly differentiated group increased more significantly (*p* < 0.01). Compared with the paracancerous regions (23083 ± 1824), the nuclear expression of β-catenin in the cancerous regions (37680 ± 3402) was upregulated (upregulated to 163% of that in the paracancerous regions, *p* < 0.001), and the cancerous regions of the moderately and poorly differentiated group was upregulated more obviously than that in the well-differentiated group (*p* < 0.001). Compared with the paracancerous regions (16.55 ± 1.04), the nucleation rate in the cancerous regions (21.35 ± 1.51) was significantly increased (upregulated to 129% of that in the paracancerous regions, *p* < 0.01), and the cancerous regions of the moderately and poorly differentiated group was increased more significantly than that of the well-differentiated group (*p* < 0.001).

We found that compared with the paracancerous regions (78445 ± 7836), the total expression of HMGB1 in the cancerous regions (97202 ± 8054) was upregulated (upregulated to 124% of that in the paracancerous regions (*p* < 0.01). Compared with the well-differentiated group, the total expression in the moderately and poorly differentiated group increased more significantly (*p* < 0.01). Compared with the paracancerous regions (8068 ± 1038), the nuclear expression of HMGB1 in the cancerous regions (16655 ± 1983) was upregulated (upregulated to 206% of that in the paracancerous regions, *p* < 0.001), and the cancerous regions of the moderately and poorly differentiated group was upregulated more obviously than that in the well-differentiated group (*p* < 0.001). Compared with the paracancerous regions (1.76 ± 0.23), the nucleation rate in the cancerous regions (3.71 ± 0.45) was significantly increased (upregulated to 211% of that in the paracancerous regions, *p* < 0.001), and the cancerous regions of the moderately and poorly differentiated group was increased more significantly than that of the well-differentiated group (*p* < 0.001; **Figures 6C, D**).

As shown in **Figures 6E, F**, compared with that in the paracancerous regions (79676 ± 8100), the expression of COX-2 in the cancerous regions (110194 ± 8022) was upregulated (upregulated to 138% of the paracancerous region, *p* < 0.01). However, COX-2 was upregulated more significantly in the moderately and poorly differentiated group than in the well-differentiated group (*p* < 0.05).

### Downregulation of aging factor p16 expression and upregulation of hypoxic factor HIF-1α expression in the cancerous regions of liver cancer patients

**Figure 7:**
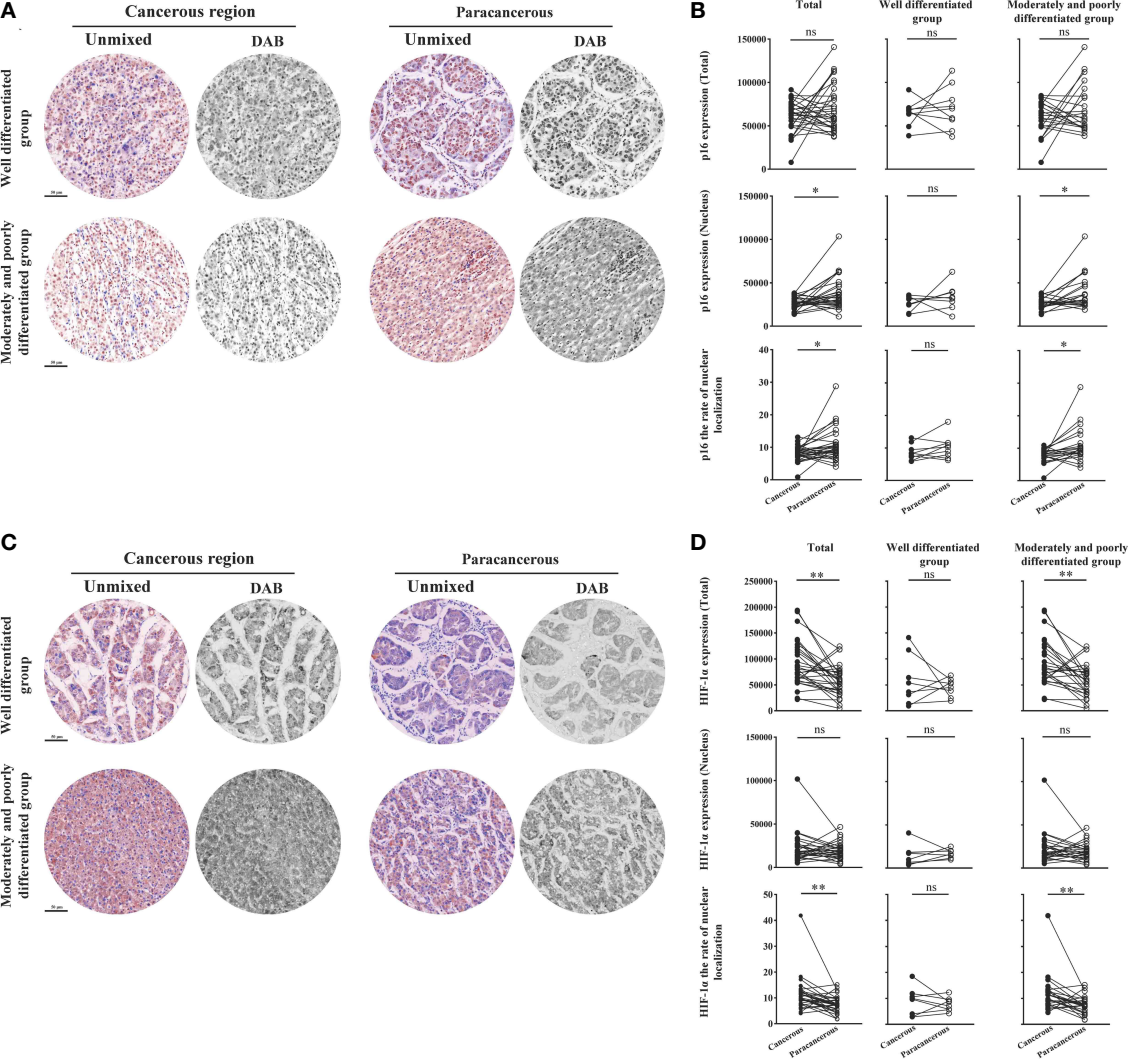
Immunohistochemical staining of p16 and HIF-1α proteins in liver cancer tissues and their quantitative images. **(A)** Representative image of immunohistochemical staining of the p16 protein in liver tissues. **(B)** Quantitative multispectral image of the p16 protein. **(C)** Representative image of immunohistochemical staining of the HIF-1α protein in liver tissues. **(D)** Quantitative multispectral image of the HIF-1α protein. The experimental data are expressed as the mean ± SEM. Compared to paracancerous regions, *p < 0.05; **p < 0.01. Scale bar, 50 µm. ns, not statistically.

**Figure 7** shows the expression levels of aging factor p16 and hypoxia factor HIF-1α in human liver cancer tissues. We found that the total expression, nuclear expression, and nucleation rate of p16 were downregulated in the cancerous regions compared to the paracancerous regions (**Figures 7A, B**). The total expression of p16 was downregulated in the cancerous regions (60204 ± 3320) compared to the paracancerous regions (70364 ± 4891; downregulated to 86% of the paracancerous regions, *p* > 0.05). However, total expression was more significantly downregulated in the moderately and poorly differentiated groups than in the well-differentiated group (*p* > 0.05). Nuclear expression of p16 was downregulated (downregulated to 71% of that in the paracancerous regions, *p* < 0.05) in the cancerous regions (26525 ± 1291) compared to the paracancerous regions (37352 ± 3442). The downregulation was more pronounced in the moderately and poorly differentiated group than in the well-differentiated group in the cancerous regions (*p* < 0.05). In addition, the nucleation rate of p16 was downregulated (downregulated to 75% of that in the paracancerous regions, *p* < 0.01) in the cancerous regions (8.06 ± 0.41) compared to the paracancerous regions (10.77 ± 0.91), and it was more significantly downregulated (*p* < 0.05) in the moderately and poorly differentiated group compared to the well-differentiated group in the cancerous regions.

As shown in **Figures 7C, D**, the total expression, nuclear expression, and nucleation rate of HIF-1α were upregulated in the cancerous regions compared to the paracancerous regions. The total expression of HIF-1α was upregulated in the cancerous regions (89486 ± 8137; upregulated to 155% of that in paracancerous regions, *p* < 0.001) compared to the paracancerous regions (57765 ± 5427), and more significantly upregulated in the moderately and poorly differentiated group compared to the well-differentiated group (*p* < 0.001). The nuclear expression of HIF-1α was not significantly different (upregulated to 114% of that in the paracancerous regions, *p* > 0.05) from that in the paracancerous regions (19158 ± 1766) compared to that in the cancerous regions (21932 ± 3184). The nucleation rate was significantly upregulated (upregulated to 150% of that in the paracancerous regions, *p* < 0.01) from that in the cancerous regions (11.43 ± 1.21) compared to that of the paracancerous regions (7.64 ± 0.60). We found that the nucleation rate was more significantly downregulated (*p* < 0.01) in the moderately and poorly differentiated group than in the well-differentiated group in the cancerous regions.

### Correlation of p-AMPK with the expression of proteins related to LKB1, mTOR, inflammatory factors (β-catenin, COX-2, HMGB1), aging factor (p16), and hypoxia factor (HIF-1α) at the same site

**Figure 8:**
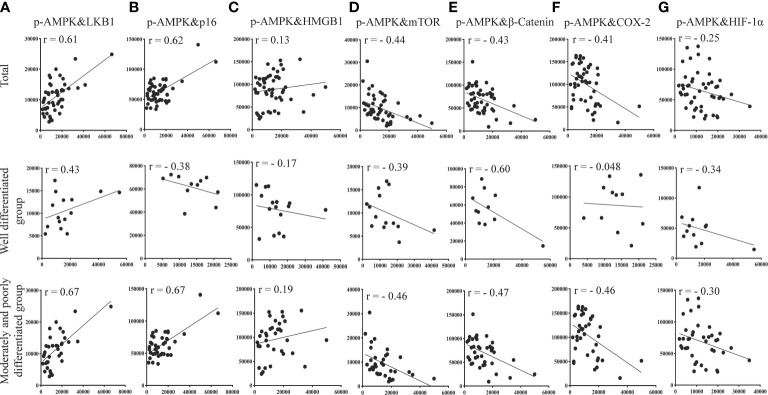
Correlation of p-AMPK with LKB1, mTOR, inflammatory factors (COX-2, β-catenin, HMGB1), aging factor (p16), and hypoxia factor (HIF-1α) proteins expressed at the same site in the same region of tumor tissue from patients with liver cancer. **(A)** Correlation between p-AMPK and LKB1 expression in the same region. **(B)** Correlation for p-AMPK and p16 expression in the same region. **(C)** Correlation of p-AMPK and HMGB1 expression in the same region. **(D)** Correlation of p-AMPK and mTOR expression in the same region. **(E)** Correlation of p-AMPK and β-catenin expression in the same region. **(F)** Correlation of p-AMPK and COX-2 expression in the same region. **(G)** Correlation of p-AMPK and HIF-1α expression in the same region. When r > 0, the two variables are positively correlated. When r < 0, the two variables are negatively correlated. When |r| ≥ 0.8, the two variables can be considered highly correlated. When 0.8 > |r| ≥ 0.5, the two variables can be considered moderately correlated. When 0.5 > |r| ≥ 0.3, the two variables can be considered slightly correlated, and |r| < 0.3 means the correlation is weak and uncorrelated.

Based on the quantitative results, we used the statistical methods of Pearson and significant difference to calculate and analyze the correlations between the expression of groups of two proteins in liver tissues (p-AMPK&LKB1, p-AMPK& mTOR, p-AMPK&β-catenin, p-AMPK&COX-2, p-AMPK&HMGB1, p-AMPK&HIF-1α, p-AMPK&p16). The results are shown in **Figure 8**. The results showed that p-AMPK was positively correlated with the expression of LKB1 (r = 0.61) and p16 (r = 0.62) and negatively correlated with the expression of mTOR (r = - 0.44), β-catenin (r = - 0.43), COX-2 (r = - 0.41), HMGB1 (r = 0.13) and HIF1α (r = - 0.25). The correlations between p-AMPK and the expression of these seven proteins in liver tissue were greater in the moderately and poorly differentiated group than in the well-differentiated group. We found a relatively high correlation between p-AMPK & LKB1, p-AMPK & p16.

## Discussion

In recent years, increasing attention has been given to the changes in the tumor microenvironment of liver cancer. It is well accepted that a fatty liver is a risk factor for liver cancer (26). In our study, we also found that the co-exposed group had more severe liver damage at the 25^th^ week than mice given a high-fat diet or nano-DEN alone. In addition, by observing the livers of mice at various periods during the experiment, we determined that severe liver steatosis is also present during the development of liver cancer. AMPK is a protein kinase that inhibits lipid production *via* phosphorylation and deactivates important adipogenic genes such as SREBP-1 (27). Further development of fatty liver leads to the formation of steatohepatitis, which is characterized by inflammation and hypoxia in the microenvironment (28). AMPK is a key master switch that regulates lipid metabolism by directly phosphorylating proteins or regulating gene transcription in specific tissues such as the liver (29).

AMPK exhibits duality in tumors, where it is rapidly activated under conditions of hypoxia and a lack of nutrients to maintain metabolic homeostasis and support the survival of cancer cells (30, 31). Therefore, activation of AMPK in the hypoxic tumor microenvironment may be the mechanism by which tumor cells enter a protective quiescent state (30). There may be a mechanism through which AMPK is increased in a compensatory manner during tumor formation and assists cancer cells in adapting to their environment. The deterioration of the tumor microenvironment, such as in inflammation and hypoxia, mediated by AMPK promoted the formation of liver cancer. We found that severe steatosis of the liver occurs during the development of liver cancer. NAFLD also becomes a metabolic dysfunction-related fatty liver disease, while it is a risk factor for HCC (32). AMPK activation reduces the formation of NAFLD (33). We found that p-AMPKα expression first increases and then decreases during liver injury, and finally, liver metabolism may be disturbed to form liver cancer due to the poor microenvironment.

Significantly more genes in the AMPK signaling pathway were discovered to be downregulated than upregulated in this study. The expression of *Prakk1* was downregulated compared to the paracancerous regions, but the expression was higher than that of the control group. Interestingly, its upstream gene *Stk11* was less expressed in cancerous regions than in the control, while the downstream gene *Mtor* was upregulated in cancerous regions. Similarly, the expression of p-AMPK and LKB1 in mouse livers first increased and then decreased during the development of liver cancer and was ultimately downregulated in cancerous regions. The expression of the mTOR was continuously increased and upregulated in cancerous regions. Research shows that, AMPK negatively regulates aerobic glycolysis in tumor cells and inhibits tumor growth *in vivo* (34). LKB1 is a tumor suppressor that is deficiently expressed in lung and breast cancers and has been identified as a key upstream kinase required for AMPK activation (35). LKB1 expression is elevated in chronic liver disease and early-stage liver cancer, and LKB1 becomes a more likely target for other posttranslational modifications in early-stage liver cancer (36).

mTOR is typically increased in malignancies, such as HCC, and is linked to a poor prognosis, poor tumor differentiation, and early recurrence (37). LKB1 has been reported to directly phosphorylate Thr172 of AMPKα *in vitro* and activate its kinase activity (38). In liver cancer, the deletion of LKB1 downregulates AMPK and promotes the expression of mTOR (34). These are consistent with our study’s findings. As a key upstream kinase that activates AMPK, LKB1 was downregulated in liver cancer, preventing AMPK activation and upregulating mTOR expression. The LKB1/AMPK/mTOR signaling pathway is aberrantly activated.

Our study showed that the expression of β-catenin, COX-2, and HMGB1 increased during the progression of liver cancer and that the expression was higher in cancerous regions than in paracancerous regions. The same was seen in human liver cancer samples. It has been found that activation of the Wnt/β-catenin signaling drives liver cancer formation and that β-catenin expression is abnormal in the majority of human liver cancer samples (34, 39). In previous studies, we confirmed the relationship of the β-catenin/COX-2 ring (40). Elevated β-catenin expression and nucleation promote COX-2 expression, and elevated COX-2 expression, in turn, promotes β-catenin expression, HMGB1 is located downstream of the β-catenin/COX-2 loop, and elevated expression of β-catenin and COX-2 promotes HMGB1 expression (40, 41). It has been found that AMPK is an upstream signal of β-catenin, and the activation of AMPK can inhibit the activity of the Wnt signaling pathway and prevent the accumulation of β-catenin in the nucleus (42, 43). Thus, the downregulation of AMPK exacerbates the involvement of the β-catenin/COX-2 loop and HMGB1 signaling in the inflammatory response. But more specific mechanisms still need to be further studied.

Protein phosphorylation is also tightly correlated with the level of oxidative stress in the cell; therefore, oxidative stress can promote phosphorylation of p16, resulting in the cessation of cell division and early senescence, thereby preventing the formation of tumors (44). The expression of p16 increases significantly with age in a variety of rodent and human tissues in both healthy and diseased states. The p16 gene has been reported to be silenced in various human tumors (45, 46). These are consistent with the results of the present study, in which we also found that p16 expression was first elevated during the formation of liver cancer and downregulated in the cancerous regions after tumor formation. Exogenous activation of LKB1/AMPK signaling increased p16 expression, and a decrease in p16 expression was observed in LKB1 knockdown cells (47, 48). These could explain the change in p-AMPK upregulation and then downregulation over time in our results. The expression of p16 was similarly upregulated and then downregulated. Inhibition of the AMPK signaling pathway resulted in the downregulation of p16 expression.

Enhanced activation of AMPK in ovarian cancer inhibits aerobic glycolysis and promotes the degradation of HIF-1α ubiquitination (49). It has been shown that in order to adapt to a hypoxic environment, tumor cells express a high level of HIF-1α, which stimulates cell metabolism and proliferation (50). It has been found that the inactivation of AMPKα promotes a metabolic shift to aerobic glycolysis and that these metabolic effects require HIF-1α to maintain stability, as silencing HIF-1α reverses the shift to aerobic glycolysis and the biosynthetic and proliferative advantages of reduced AMPKα signaling in tumor cells (51). Therefore, the high expression of HIF-1α in the development of liver cancer is influenced by AMPK.

There are many studies that have demonstrated the close relationship between AMPK and β-catenin, p16 and HIF-1α, respectively. However, there may be a mutual relationship between them. We will conduct a deeper study next.

## Conclusion

In summary, we can conclude that fatty liver deteriorates into liver cancer due to AMPK-mediated metabolic disorders, cellular aging and chronic inflammation in the liver. Malignant alterations in the tumor microenvironment inhibit the AMPK signaling pathway, resulting in a disruption of energy metabolism, chronic inflammation, fat deposition, and cellular aging. Promotes the progression of fatty liver to cancer of the liver.

## Data availability statement

The datasets presented in this study can be found in online repositories. The names of the repository/repositories and accession number(s) can be found below: https://db.cngb.org/cnsa/, CNP0003645.

## Ethics statement

The studies involving human participants were reviewed and approved by the Committee on the Ethics of Experiments of South-Central Minzu University in China (Permit Number: 2017-SCUEC-MEC-007). Written informed consent for participation was not required for this study in accordance with the national legislation and the institutional requirements. The animal study was reviewed and approved by the Laboratory Animal Society of the Research Facilities Committee. All research on mice was approved by the Animal Experiment Ethics Committee of South-Central Minzu University, Wuhan, China (permit number: 2018-SCUEC-AEC-010).
