# Supplementary material for: Gradual deterioration of fatty liver disease to liver cancer via inhibition of AMPK signaling pathways involved in energy-dependent disorders, cellular aging, and chronic inflammation
Source: Front Oncol. 2023 Mar 2;13:1099624. doi: 10.3389/fonc.2023.1099624 (PMC10018212; doi:10.3389/fonc.2023.1099624)
Supplement: Supplementary file 1 [file DataSheet_1.doc]

A folder named "Raw Data" containing Pictures.pptx and original data.xlsx has been uploaded to Nutstore. Please check its data link (https://www.jianguoyun.com/p/DbWxP40Ql8qVCxjd2-kEIAA).
